# Role of Corticosterone on Sleep Homeostasis Induced by REM Sleep Deprivation in Rats

**DOI:** 10.1371/journal.pone.0063520

**Published:** 2013-05-07

**Authors:** Ricardo Borges Machado, Sergio Tufik, Deborah Suchecki

**Affiliations:** Psychobiology Department, Universidade Federal de São Paulo, São Paulo, Brazil; Morehouse School of Medicine, United States of America

## Abstract

Sleep is regulated by humoral and homeostatic processes. If on one hand chronic elevation of stress hormones impair sleep, on the other hand, rapid eye movement (REM) sleep deprivation induces elevation of glucocorticoids and time of REM sleep during the recovery period. In the present study we sought to examine whether manipulations of corticosterone levels during REM sleep deprivation would alter the subsequent sleep rebound. Adult male Wistar rats were fit with electrodes for sleep monitoring and submitted to four days of REM sleep deprivation under repeated corticosterone or metyrapone (an inhibitor of corticosterone synthesis) administration. Sleep parameters were continuously recorded throughout the sleep deprivation period and during 3 days of sleep recovery. Plasma levels of adrenocorticotropic hormone and corticosterone were also evaluated. Metyrapone treatment prevented the elevation of corticosterone plasma levels induced by REM sleep deprivation, whereas corticosterone administration to REM sleep-deprived rats resulted in lower corticosterone levels than in non-sleep deprived rats. Nonetheless, both corticosterone and metyrapone administration led to several alterations on sleep homeostasis, including reductions in the amount of non-REM and REM sleep during the recovery period, although corticosterone increased delta activity (1.0–4.0 Hz) during REM sleep deprivation. Metyrapone treatment of REM sleep-deprived rats reduced the number of REM sleep episodes. In conclusion, reduction of corticosterone levels during REM sleep deprivation resulted in impairment of sleep rebound, suggesting that physiological elevation of corticosterone levels resulting from REM sleep deprivation is necessary for plentiful recovery of sleep after this stressful event.

## Introduction

Sleep homeostasis is regulated by humoral, circadian and homeostatic factors. Among the humoral factors, stress hormones are of great importance, given the negative influence that certain forms of chronic stress have on sleep, both in humans [1], [2], [3] and animals [4], [5], [6], [7]. For instance, corticotropin-releasing hormone (CRH), the main triggering neuropeptide of the hypothalamic-pituitary-adrenal (HPA) axis, is a major regulator of waking, and inhibits non-REM (NREM) sleep by acting both at the hypothalamic and extra-hypothalamic levels [8], [9], [10], [11], and REM sleep even in REM sleep deprived rats [12] In regard to the effects of corticosterone on sleep, administration of high doses of corticosterone increases sleep latency, waking time after sleep onset and number of awakening episodes [13], [14], and reduces the time of NREM sleep [15], whereas inhibition of corticosterone synthesis, by acute administration of metyrapone, suppresses both REM and non-REM sleep [16], [17], [18], [19]. Because metyrapone inhibits 11-β-hydroxilase, the enzyme that converts 11-deoxicortisol/11-deoxicorticosterone to cortisol/corticosterone [20] there is a reduction of corticosterone negative feedback at the hypothalamic and pituitary levels, resulting in increased CRH activity. Adrenalectomy, likewise, reduces the potency of lower frequency bands (1.0 to 4.0 Hz) and increases the potency of higher ones (9.0 to 12.0 Hz), which is promptly reversed by corticosterone supplementation [15].

Regarding the homeostatic regulation, it is manifested after long periods of forced awakening, after which a period of compensatory sleep ensues, with augmented NREM and REM sleep [21], [22]. In rodents, brief (3 to 6 h) periods of total sleep deprivation result in rebound of NREM sleep [23], whereas longer periods (12 to 24 h) result in REM sleep rebound [24], [25]. Four days of unremitting REM sleep-deprivation (REMSD) produces a specific rebound of REM sleep, and negligible rebound of NREM [26], [27], most likely because this method allows NREM sleep to take place [26]. In addition, long periods of total sleep deprivation may also results either in no or negative (under basal levels) NREM sleep rebound (see [28] for review). Changes in sleep microarchitecture, involving both low and high frequencies bands, have been reported after total or partial sleep deprivation procedures, in humans [29], [30], [31] and animals [25], [32], [33], [34].

Recently, we showed that physiological elevation of corticosterone in REM sleep-deprived rats exposed to stress during the deprivation period, has modulatory effects on sleep, inasmuch as intermediate levels favors the expression of REM sleep rebound, whereas low or high levels impair sleep rebound [12]. CRH administration, on the contrary, inhibits REM sleep rebound, even in REM sleep-deprived rats that are prone to exhibit this phenomenon [35]. Given that in rats REM sleep deprivation activates the hypothalamic-pituitary-adrenal (HPA) axis [36], [37], [38] and that stress hormones, in turn, modulate sleep, in the present study we evaluated the outcomes of manipulating corticosterone levels (by chronically treating different groups of rats with metyrapone or corticosterone) throughout a protocol of forced wakefulness on sleep macro-and microstructure in rats. ACTH and corticosterone plasma concentrations were determined as a means to ensure that the pharmacological manipulations produced the expected neuroendocrine effects.

## Methods

### Ethics Statements

The study protocol was approved by the Research Ethics Committee of the Universidade Federal de São Paulo (CEP 0125/04) in accordance with international guidelines for care in animal research.

### Subjects

Male adult Wistar rats (350–450 g) from our own animal facility were used (eight to ten animals per group). A constant 12 h light-dark cycle (fluorescent white lamps-lights on at 7:00 h) and temperature (22±2°C) was maintained in all experimental rooms throughout the experimental protocol. Animals had free access to food and water during the entire study.

### Electrophysiological procedures

Electrodes to monitor the sleep-wake cycle were implanted under ketamine + xylazine anesthesia (Dopalem® and Anasedan®, Vetbrands, Brazil; 90.0 and 10.5 mg/kg, i.p., respectively): two bipolar electrodes placed ipsilaterally with stainless-steel micro-screws (316 nickel-chromium alloy, generic manufacturer, Brazil; 0.9 mm of diameter and of 2.0 mm of length) were used for EEG monitoring: one pair in the right lateral parieto-parietal (for minimum theta activity EEG) and the other, in the left medial fronto-parietal (for maximum theta activity EEG) areas [39], [40]. One pair of insulated nickel-chromium flexible fine wire electrodes (California Fine Wire®, USA) was implanted in the dorsal neck muscle for EMG recording. After the surgical procedure, a broad spectrum antibiotics association (Pentabiótico®, Fort-Dodge, Brazil) and sodium diclofenac (Voltarem®, Novartis, Brazil) were injected, intra-muscle, and the animal was allowed to recover from surgery for 15 days.

Animals were habituated to the recording cables and to the Faraday's chambers for 3 days before baseline sleep recording, which was performed for two consecutive days (2×24 h) and the values presented were obtained by averaging these two days. After the baseline recording, in the period that preceded REMSD, animals were adapted to the sleep deprivation chambers for 30 minutes per day for three consecutive days. Animals were continuously recorded during the sleep deprivation and recovery periods.

Electrophysiological signals were recorded on a digital polygraph (Neurofax QP 223A, Nihon Kohden, Japan). After conventional amplification, the EEG signals were conditioned through analogical filters, using cut off frequencies of 1.0 Hz and 35.0 Hz, and were then sampled at 200 Hz using a 16 bits A/D converter. Recordings were displayed on 10 s epochs and submitted off-line to visual scoring routine, as described previously [41]. The following parameters were compared within each (light or dark, separately) recording period throughout the study: Total sleep time (percentage of time spent in sleep during the corresponding recorded period); non REM (NREM) sleep–percentage of time spent in NREM phase during the each of the 12 h recording period. In rats, as in humans, NREM sleep is not a homogeneous state, therefore, in the present study, it was sub-classified in low amplitude, LNREM (EEG amplitude varies between 20.0 and 30.0 µV) and high amplitude, HNREM (average of EEG voltage above 30.0 µV, because in rodents, the EEG amplitude is the main hallmark [39], [42], [43]); REM Sleep – percentage of time spent in REM sleep throughout the recording time; Number of REM episodes (sum of all events of REM sleep); Mean Length of REM Episodes (average duration, in minutes, of REM sleep bouts during each 12 h recording period); Bouts of waking (number of bouts of waking longer than 2.0 min.). Waking periods were also divided in quiet wake (QW) and active wake (AW), mainly as a function of EMG activity.

Fast Fourier Transform (Hanning window) was computed on 256 points (corresponding to each vigilance state) with a resolution of 0.78 Hz. Non-overlapping bands were set giving 0.5 Hz bins from 1.0 to 5.0 Hz, and 1.0 Hz bins from 5.1 Hz to 25.0 Hz, and those above 25.0 Hz were discarded from analysis. EEG epochs containing noise or artifacts were excluded from the analysis by visual inspection and/or spectral tools (e.g. if average power exceeded 2000 µVolts^2^ over a 1.0 25.0 Hz frequency range). Slow Wave Activity-SWA was calculated as mean power density on 1.0–4.0 Hz (delta band) and the Accumulated Slow Wave Activity-ASWA reflects the sum of total SWA occurring during all NREM sleep episodes in each 12 h recording period. The lateral parieto-parietal deviation was used for NREM sleep EEG spectral analysis due its high delta activity and good correlation with sleep phases [39], [40].

### Sleep deprivation procedure and drug administration

That was accomplished by the single platform method, in which the animal is placed, individually, onto a narrow cylindrical platform, 6.5 cm in diameter surrounded by water about 1 cm below the platform surface. This method is well known for producing selective REM sleep-deprivation and reduction of non-REM sleep [26], [44]. Twice a day (at 7:00 h and 19:00 h) during the four days of sleep deprivation and immediately after the end of the deprivation period (at 7:00 h), corticosterone (crystalline, Sigma, USA; 5 mg/kg; s.c.; finely pulverized and suspended in corn oil) or metyrapone (powder, Aldrich, Germany; 100 mg/kg; i.p., diluted in warm propyleneglycol) were administered, making up for nine administrations. Different solvents were chosen based on their compatibility with the drugs and their low toxicity for the animals, and different administration routes also were chosen according with best pharmacokinetics drug properties for the desired effects (e.g. increase or decrease on plasma corticosterone, obtained from preliminary pilot studies). The sleep pattern of these two groups was compared to that of REM sleep-deprived rats treated with sterile saline (1 mL/kg; i.p.) under the same schedule. After four days of sleep deprivation, rats were allowed to sleep freely in their individual home cages (recovery period) for three days.

### Blood hormones

**Figure 1:**
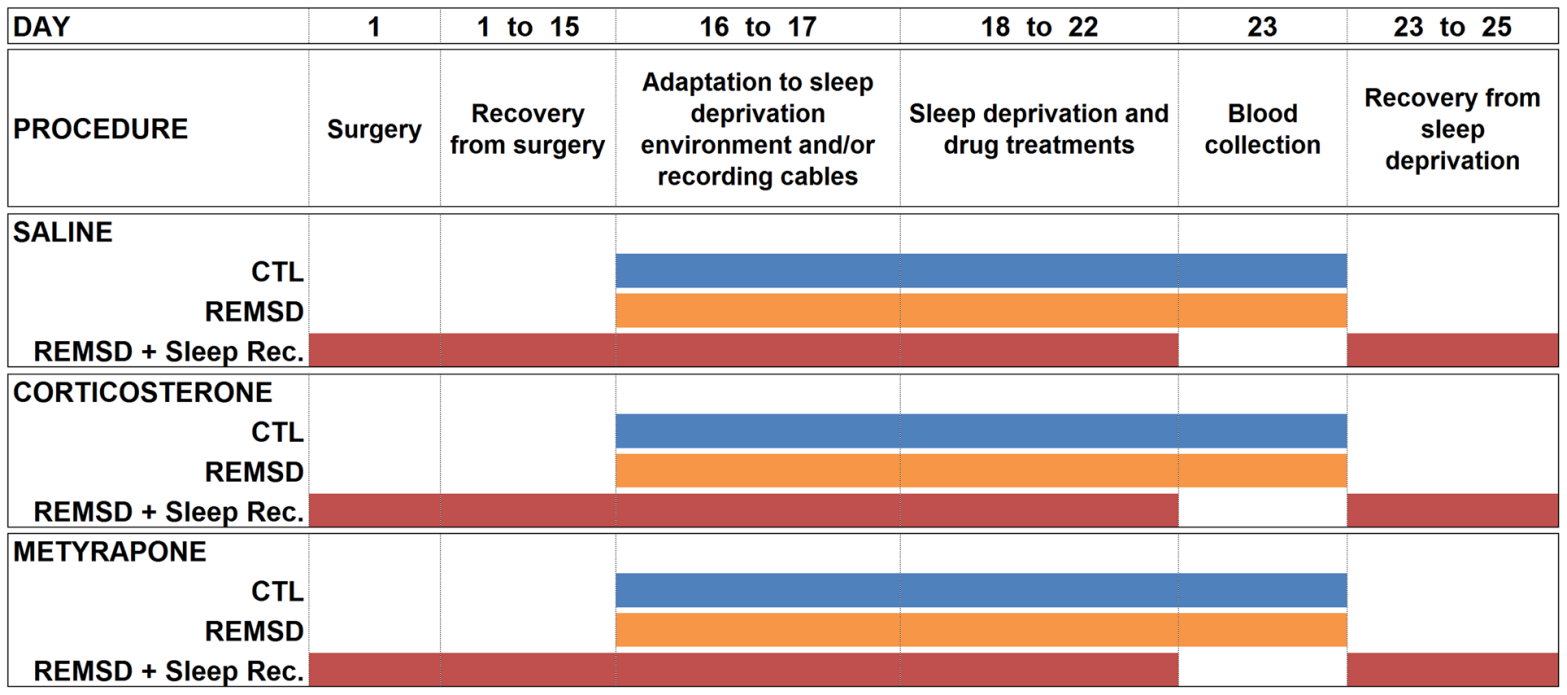
Experimental Design. Colorful bars indicate the procedure to which each group was submitted within treatments. CTL, home-cage control; REMSD, REM sleep deprivation; Sleep Rec, Sleep recovery.

Trunk blood was obtained by decapitation approximately 2 h after the last injection, from matched groups, run simultaneously with the sleep study (see Fig. 1). During these 2 h, animals were prevented from sleeping, by being placed back into the deprivation chambers. Blood was collected in chilled vials containing K_2_EDTA (0.46 mM) and centrifuged at 2300 rpm, at 4°C for 20 min; plasma was collected and frozen for further analysis. Plasma ACTH was determined by sequential immunometric assay (DPC Immulite, USA) and corticosterone levels, by specific radioimmunoassay (INC Biomedicals, USA). Endogenous and exogenous corticosterone were not differentiated, given the non specificity of the antibody employed. All assays were performed in duplicate.

### Statistical analysis

General analysis of Hormone levels were done by the General Linear Model with a two-way factorial ANOVA, with main factors Group (Home-cage control [CTL] and REM sleep-deprivation [REMSD]) and Treatment (Saline [SAL], Corticosterone [CORT], Metyrapone [MET]). For the sleep parameters, data were analyzed in two steps: the first one to compare different days of REM sleep deprivation with baseline sleep and the second one, different days of recovery with baseline sleep. Data was analyzed separately for light and dark phases (12 h analysis blocks), by a two-way ANOVA for repeated measures, with main factors Treatment (SAL, CORT, MET) and Days (repeated measure: Baseline, REM sleep-deprivation days [Dep 1, Dep 2, Dep 3 and Dep 4]; and Baseline, Recovery days [Rec 1, Rec 2 and Rec 3]). *Post-hoc* analyses were performed by the Newman-Keuls test for factorial ANOVA and Test of Bonferroni for repeated ANOVA. The level of significance was set at *p*≤0.05.

## Results

### Hormones of the HPA axis

#### ACTH

**Figure 2:**
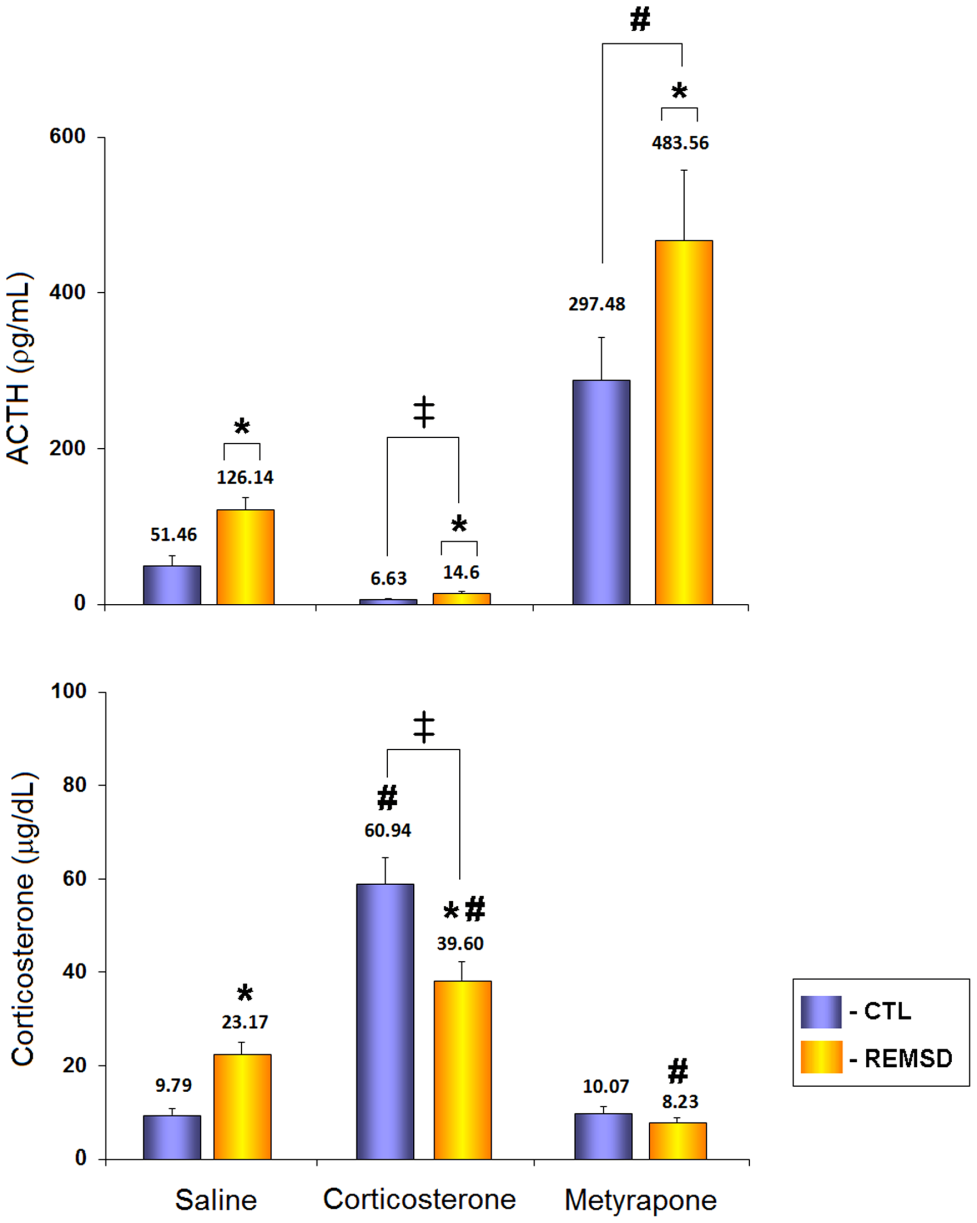
ACTH and Corticosterone Plasma Levels in Response to Treatments with Corticosterone or Metyrapone. CTL, home-cage control; REMSD, REM sleep deprivation. Data is presented as mean ± S.E.M. of 8–10 rats/group. ⋆ Different from respective CTL groups; # different from SAL-treated rats; ‡ different from MET-treated rats; lines above bars indicate main effects (group or treatment). ANOVA followed by Newman-Keuls test; *p*≤0.05.

Main effects of group (F_1,50_ = 6.23, *p*≤0.05) and treatment (F_2,50_ = 38.86, *p*≤0.00001) were observed. REMSD increased ACTH levels compared to CTL animals (77.84%, *p*≤0.02). In respect to treatment, CORT-treated rats displayed lower levels of ACTH (85.10%, *p*≤0.05), whereas MET-treated animals secreted more ACTH (333.76%, *p*≤0.0002) than SAL-treated animals (Fig. 2).

#### Corticosterone

Main effects of treatment (F_2,52_ = 97,95; p≤0.00001) and an interaction between treatment and group (F_2,52_ = 15.01, *p*≤0.00005) were shown. Analysis of the interaction revealed that SAL-treated REMSD rats secreted more CORT than their respective CTL group (136.61%, *p*≤0.01). Conversely, REMSD rats treated with corticosterone displayed lower CORT levels than their respective CTL group (−35.01%, *p*≤0.0005), whereas no difference between these groups was seen with metyrapone. Comparison of the treatments within groups showed that for CTL rats, CORT treatment resulted in higher CORT levels (204.99%, *p*≤0.0005), whereas there was no difference between MET- and SAL-treated rats. As for REMSD rats, CORT treatment increased, whereas MET decreased CORT plasma levels, compared to SAL-treated rats (−44.49%, p≤0.001) (Fig. 2).

### Sleep Parameters (only REM sleep-deprived animals)

#### Total Sleep Time

**Figure 3:**
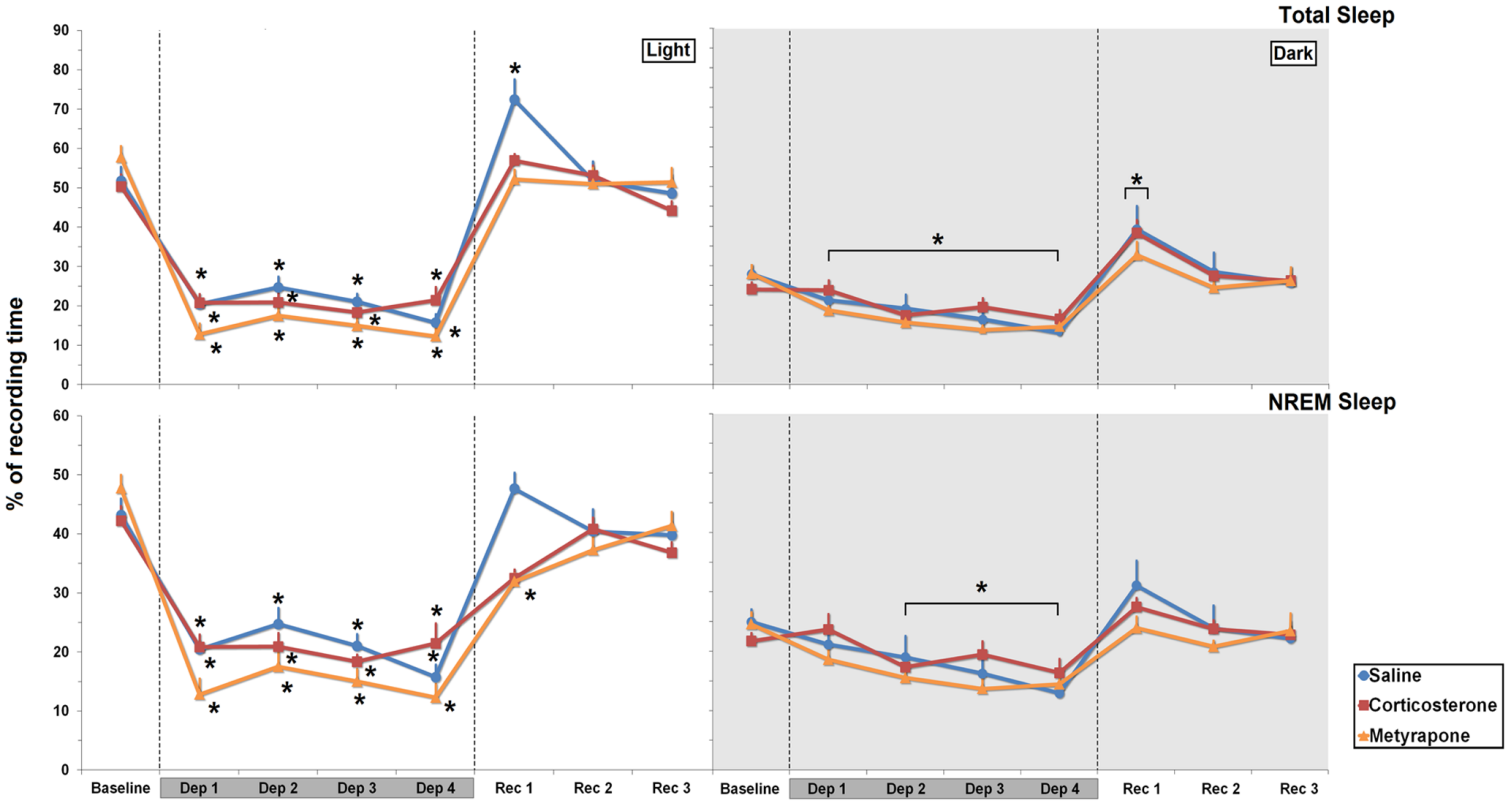
Total Sleep Time and NREM Sleep. Results are expressed as percentage of recording time (≅12 h), in SAL-, CORT- and MET-REM sleep-deprived treated rats, during light and dark phases. Data is presented as mean ± S.E.M. of 8–10 rats/group. Dep: REM sleep deprivation; Rec: recovery period. Dark-grey bars over the days indicate the sleep deprivation period. The hatched lines indicate the different phases of the experimental procedure. ✶ Different from baseline. Main effect of day is indicated by brackets above the symbols. Further differences can be found in the Results session. ANOVA followed by Bonferroni test, *p*≤0.05.

Light phase: During the deprivation period, there were a main effect of Day (F_4,84_ = 149.84, *p*≤0.000001) and an interaction between Day and Treatment (F_8,84_ = 3.009, *p*≤0.006). Analysis of this interaction with Test of Bonferroni revealed a reduction of percentage of time spent sleeping (*p*≤0.00001). Analysis of the recovery period showed main effect of Day (F_3,63_ = 7.993; *p*≤0.0002) and an interaction between Day and Treatment (F_6,63_ = 3.841; *p*≤0.003). *Post hoc* analysis indicated that only SAL-treated rats slept more in the first recovery day than baseline (within-group comparison: 39.96%, *p*≤0.0003) (Fig. 3).

*Dark phase*: A main effect of Day was observed (F_4,84_ = 13.895, *p*≤0.000001); compared to baseline sleep, total sleep was reduced throughout the sleep deprivation period (−34.32%, *p*≤0.05). Analysis of the recovery period showed a main effect of Day (F_3,63_ = 11.724; *p*≤0.000003), and all groups slept more in the first recovery night than in the baseline (38.11%, *p*≤0.0001) (Fig. 3).

#### NREM Sleep

*Light phase*: There was a main effect of Day (F_4,84_ = 99.086, *p*≤0.000001) and an interaction between Treatment and Day (F_8,84_ = 2.894, *p*≤0.007) for the comparison between REM sleep deprivation period and baseline. Pairwise comparisons with the test of Bonferroni showed a reduction of percentage of NREM sleep during the deprivation period, for all rats, regardless of treatment (*p*≤0.00001). Comparison of recovery days and baseline sleep revealed a main effect of Day (F_3,63_ = 4.765; *p*≤0.005) and an interaction between Day and Treatment (F_3,63_ = 4.411; *p*≤0.001). MET-treated rats was the only group that showed negative NREM rebound (below baseline levels) on REC 1 (−33.08%, *p*≤0.001) (Fig. 3).

*Dark phase*: A main effect of Day was observed (F_4,84_ = 9.265, *p*≤0.000003); for all treatments NREM sleep was reduced on the 2^nd^, 3^rd^ and 4^th^ nights of sleep deprivation (−27.08%, *p*≤0.004; 30.57%, *p*≤0.0007 and −38.47% *p*≤0.000009, respectively). Analysis of the recovery period showed a main effect of Day (F_3,63_ = 3.949; *p*≤0.02), with greater percentage of NREM in the first recovery day, compared to REC 2 and REC 3 (20.47% and 20.57%, respectively; *p*≤0.04) (Fig. 3).

#### Slow Wave Activity during NREM Sleep

**Figure 4:**
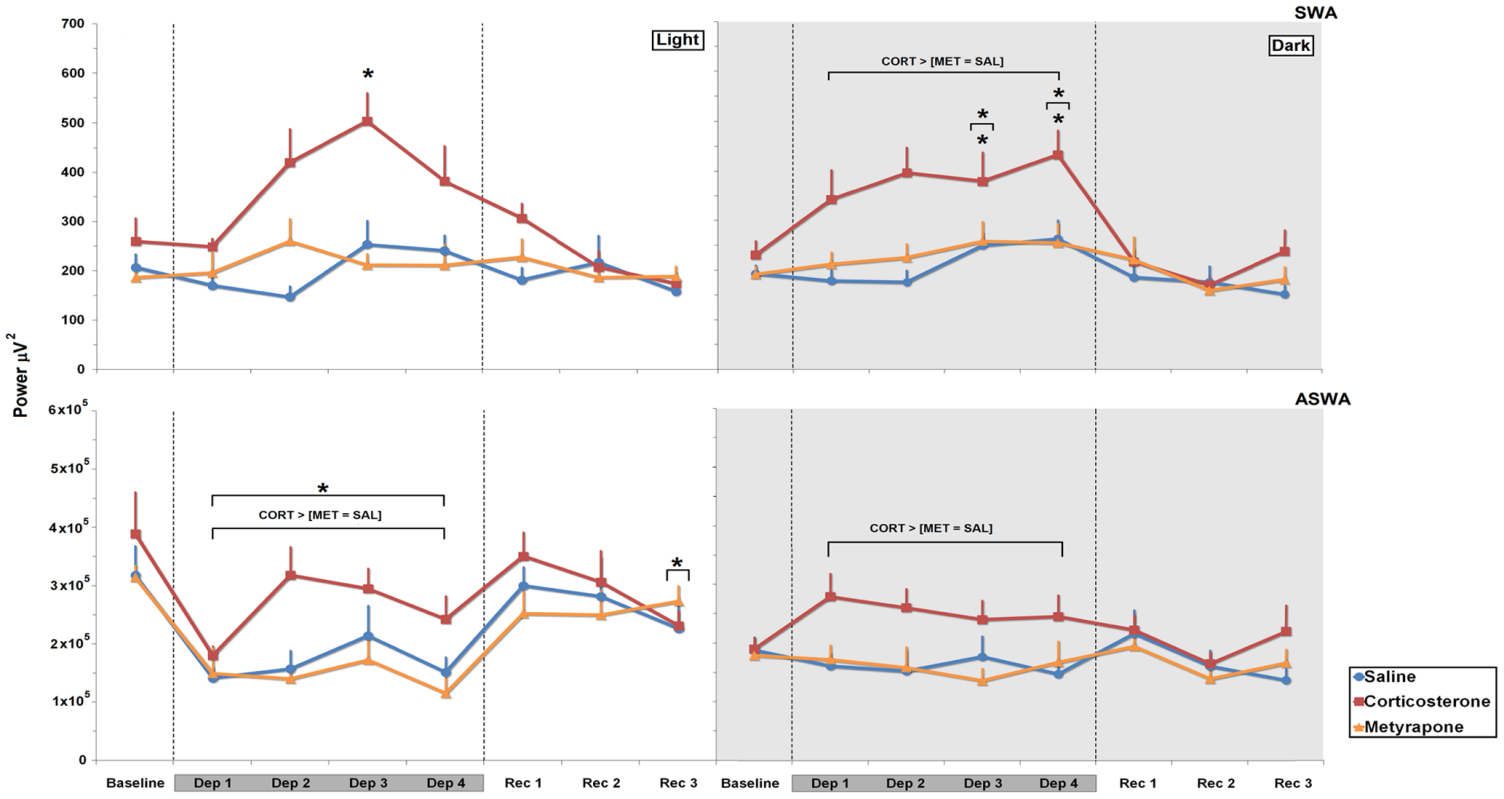
Slow Wave Activity (SWA) and Accumulated Slow Wave Activity (ASWA) in NREM Sleep. Slow Wave Activity (1.0–4.0 Hz), expressed as mean power density (micro-volts squared-µV^2^) of the lateral parietoparietal EEG and the Accumulated Slow Wave Activity-ASWA reflects the sum of total SWA occurring during all NREM sleep episodes, pooled at every ≅12 h recording period, in SAL-, CORT- and MET-REM sleep-deprived treated rats, during light and dark phases. Mean ± S.E.M. of 8–10 rats/group. Dep: REM sleep deprivation; Rec: recovery period. Dark-grey bars over the days indicate the sleep deprivation period. The hatched lines indicate the different phases of the experimental procedure. ⋆ Different from baseline, # different from SAL-treated rats, ‡ different from MET-treated rats. Further differences can be found in the Results session. Main effects of day or treatment are indicated by brackets above the symbols. ANOVA followed by Bonferroni test, *p*≤0.05.

*Light phase*: There were main effects of Treatment (F_2,21_ = 9.296, *p*≤0.002), Day (F_4,84_ = 6.418, *p*≤0.0002) and an interaction between these factors (F_8,84_ = 3.468, *p*≤0.002). Analysis of this interaction showed that in CORT-treated animals NREM slow wave activity was higher on the 3^rd^ day of the sleep deprivation period than baseline (94.05%, *p*≤0.0002). In addition, SWA was higher on day 2 and 3 than on day 1 of REM sleep deprivation (68.78%, *p*≤0.05 and 102.63%, *p*≤0.0.0001) in this same group. No differences among the treatments were observed during the sleep recovery period (Fig. 4).

*Dark phase*: There were main effects of Treatment (F_2,20_ = 8.29, *p*≤0.003), Day (F_4,80_ = 6.896, *p*≤0.0001) but no interaction between these factors regarding the sleep deprivation period. Post hoc analysis indicated that during sleep deprivation, NREM SWA increased in CORT-treated animals when compared to SAL- and MET-treated rats (68.22%, *p*≤0.004 and 53.50%, *p*≤0.02). NREM SWA was augmented on 3^rd^ and 4^th^ days of sleep deprivation compared to baseline (64.37%, *p*≤0.002; 87.63%, *p*≤0.00008); the SWA levels were also higher on the 4^th^ day than on the 1^st^ day of sleep deprivation (28.63%, *p*≤0.04). Again, no differences were observed during the sleep recovery period (Fig. 4).

#### Accumulated Slow Wave Activity during NREM Sleep

*Light phase*: Main effects of Treatment (F_2,21_ = 6.286, *p*≤0.008) and Day (F_4,84_ = 8.75, *p*≤0.000001) were detected for the sleep deprivation period. Regarding the Treatment effect, in CORT-treated animals ASWA was 31.27% higher than SAL- (*p*≤0.04) and 41.82% higher than MET-treated rats (*p*≤0.01). As for the effect of Day, during REM sleep-deprivation the animals displayed less ASWA than their respective baselines (−46.34%, in average, *p*≤0.005). As for the recovery period, there was a main effect of Day (F_3,63)_ = 2.759; *p*≤0.05), with a significant reduction on the last recovery day when compared to baseline (−29.74%, *p*≤0.04) (Fig. 4).

*Dark phase:* A main effect of Treatment was observed during REM sleep deprivation (F_2,21_ = 5.932, *p*≤0.009) and CORT-treated rats exhibited more ASWA than SAL- (42.69%, *p*≤0.03) and MET-treated rats (46.07%, *p*≤0.02). No differences among groups were observed during the recovery period (Fig. 4).

#### REM Sleep

Due to the complete suppression of REM sleep induced by the platform technique, analysis of the data included only baseline sleep and the recovery period.

**Figure 5:**
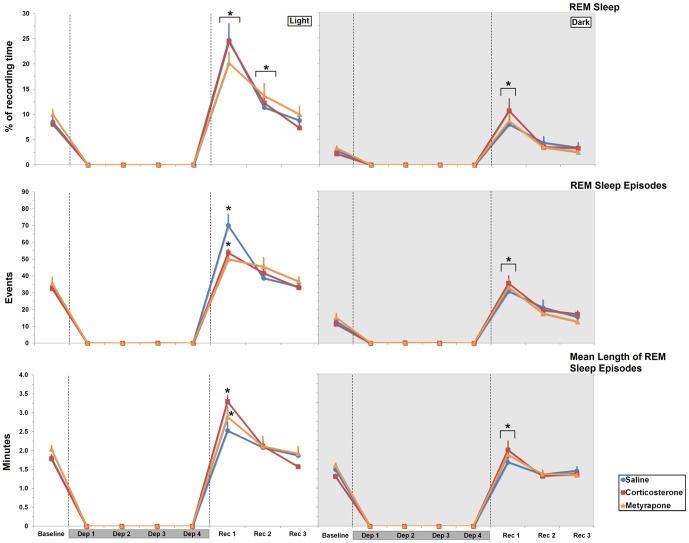
REM Sleep, REM Sleep Episodes and Mean Length of REM Sleep Episodes. REM Sleep results are expressed as percentage of recording time (≅12 h), in SAL-, CORT- and MET-REM sleep-deprived treated rats, during light and dark phases. REMS Episodes were expressed as absolute number and Mean length of REM Episodes were expressed in minutes. Mean ± S.E.M. of 8–10 rats/group. Dep: REM sleep deprivation period; Rec: recovery period. Dark-grey bars over the days indicate the sleep deprivation period. The hatched lines indicate the different phases of the experimental procedure. ⋆ Different from baseline. Main effect of day is indicated by brackets above the symbols. ANOVA followed by Bonferroni test, *p*≤0.05.

*Light phase*: The two-way ANOVA for repeated measures detected a main effect of Day (F_3,63_ = 63.577, *p*≤0.00001), in which the animals showed increased REM during Rec 1 and Rec 2 compared to baseline (160.88%, *p*≤0.00001; 40.47%, *p*≤0.03; respectively) (Fig. 5).

*Dark phase*: Again, a main effect of Day was observed (F_3,63_ = 27.55, *p*≤0.00001), with increased REM sleep (*p*≤0.0005) only on Rec 1 when compared to baseline levels (Fig. 5).

#### Number of REM Sleep Episodes

*Light phase*: A main effect of Day (F_3,63_ = 29.69, *p*≤0.000001) and an interaction between Treatment and Day was detected (F_6,63_ = 3.045, *p*≤0,02). Within-group comparisons revealed that SAL- and CORT-treated animals exhibited more REM sleep events during Rec 1 than baseline (107.78%, *p*≤0.00005 and 65.45%; *p*≤0.01, respectively). No differences were found among the groups (Fig. 5).

*Dark phase*: A main effect of Day was revealed (F_3,63_ = 27.55, *p*≤0.00001) and the rats displayed more REM sleep events during Rec 1 (151.27%, *p*≤0.00001) than baseline sleep (Fig. 5).

#### Mean Length of REM Sleep Episodes

*Light phase*: An interaction between Treatment and Day was revealed (F_6,63_ = 2.193, *p*≤0.05); Bonferroni test showed that both CORT- and MET-treated groups exhibited longer REM sleep episodes in Rec 1 than baseline (CORT: 85.14%, *p*≤0.000001; MET: 41.67%, *p*≤0.03). There were no between group differences (Fig. 5).

*Dark phase*: A main effect of Day was observed (F_3,57_ = 10.75, *p*≤0.00005) and all groups exhibited longer REM sleep episodes in Rec 1 than baseline (27.12%, *p*≤0.0007), than Rec 2 (37.24%, *p*≤0.00002) and than Rec 3 (30.52%, *p*≤0.0003) (Fig. 5).

#### Number of awakenings

**Figure 6:**
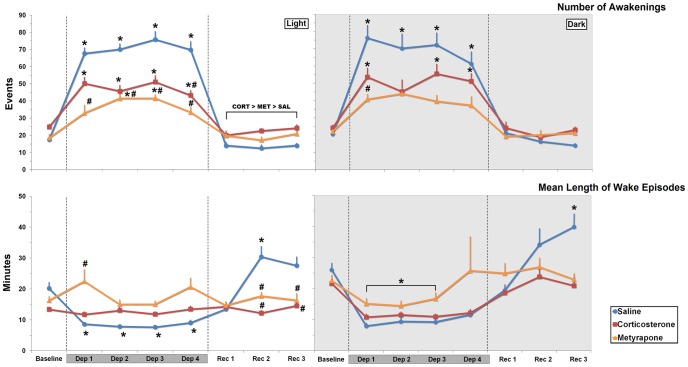
Episodes of awakening and Mean Length of Wake Episodes. Results of Episodes of Awakening longer than 2.0 min. are expressed as absolute number and Mean Length of Wake Episodes, in minutes, during the light and dark phases, in SAL-, CORT- and MET-REM sleep-deprived treated rats. Mean ± S.E.M. of 8–10 rats/group. Dep: REM sleep deprivation period; Rec: recovery period. Dark-grey bars over the days indicate the sleep deprivation period. The hatched lines indicate the different phases of the experimental procedure. Main effects of day or treatment are indicated by brackets above the symbols. ⋆ Different from baseline, # different from SAL-treated rats. ANOVA followed by Bonferroni test, *p*≤0.05.

*Light phase*: There was a main effect of Treatment (F_2,21_ = 47.51, *p*≤0.000001), of Day (F_4,84_ = 65.533, *p*≤0.000001) and an interaction between these factors (F_8,84_ = 8.248, *p*≤0.000001). Analysis of this interaction revealed that awakenings longer than 2.0 min were higher during all days of sleep deprivation than baseline for SAL- and CORT-treated rats (average, SAL: 303.66%, *p*≤0.000001; CORT: 90.89%, *p*≤0.01). For MET-treated rats, increased number of awakenings above baseline occurred on Dep 2 and Dep 3 (average, 124.64%, *p*≤0.0001). Throughout the deprivation period, MET-treated rats exhibited fewer awakening events than SAL-treated rats (−47.59%, *p*≤0.02). CORT-treated animals showed less awakening events on Dep 4 when compared with SAL-treated rats (−38.05%, *p*≤0.04). Analysis of the sleep recovery period showed a main effect of Treatment (F_2,21_ = 17.335, *p*≤0.0001). Post-hoc analysis revealed that CORT-treated rats had more awakening events than SAL-treated (58.32%, *p*≤0.00003) and than MET-treated rats (16.73%, *p*≤0.04), which, in turn had more events than SAL-treated ones (31.82%, *p*≤0.01) (Fig. 6).

*Dark phase*: Main effects of Treatment (F_2,21_ = 20.328, *p*≤0.00002), Day (F_4,84_ = 24.713, *p*≤0.000001) and an interaction between these factors (F_8,84_ = 2.635, *p*≤0.02) were shown throughout the deprivation period. SAL- and CORT-treated rats displayed more awakenings longer than 2.0 min (SAL: 240.58%, *p*≤0.00002; CORT: 112.47%, *p*≤0.03 [except during the 2^nd^ deprivation day]). Between-group analysis indicated that MET-treated rats displayed fewer awakenings during the 1^st^ day of sleep deprivation than SAL-treated rats (46.6%, *p*≤0.04). No differences among groups were observed during the recovery period (Fig. 6).

#### Mean Length of wake episodes

*Light phase*: There was a main effect of Treatment (F_2,21_ = 15.515, *p*≤0.0001), Day (F_4,84_ = 6.428, *p*≤0.0002) and an interaction between these factors (F_8,84_ = 7.099, *p*≤0.000001). Within-group comparisons showed that only SAL-treated rats displayed shorter wake episodes during sleep deprivation period (average −58.84%, *p*≤0.0001). Comparison among the groups indicated that MET-treated rats displayed longer wake episodes in the 1^st^ day of deprivation Dep 1 than SAL-treated ones (160.05%, *p*≤0.02). During the recovery period main effects of Treatment (F_2,21_ = 21.386, *p*≤0.0001), Day (F_3,63_ = 8.246, *p*≤0.0001) and an interaction between these factors (F_6,63_ = 6,899, *p*≤0.00002). Only SAL-treated rats exhibited longer wake episodes on the 2^nd^ day of recovery (50.68%; *p*≤0.004) than baseline. Moreover, both CORT- and MET-treated rats showed shorter wake episodes in Rec 2 (−59. 85%, *p*≤0.0003 and −47.12%, *p*≤0.02, respectively) and Rec 3 for CORT-treated animals (−41.79%, *p*≤0.02) than SAL-treated animals (Fig. 6).

*Dark phase*: There was a main effect of Day (F_4,76_ = 7.015, *p*≤0.0001); shorter episodes were displayed on Dep 1, Dep 2 and Dep 3 (−50.54%, *p*≤0.0002, −49.01%, *p*≤0.0004 and −45.55%, *p*≤0.001, respectively) than baseline. During the recovery period, ANOVA revealed main effects of Treatment (F_2,21_ = 6.056, *p*≤0.009), Day (F_3,63_ = 5.918, *p*≤0.002) and an interaction between these factors (F_6,63_ = 4.029, *p*≤0.002). Longer wake episodes were found in SAL-treated rats, on the third recovery day, than their baseline (53.33%, *p*≤0.02) (Fig. 6).

## Discussion

The method used to produce sleep deprivation completely suppressed REM sleep and significantly reduced NREM sleep; as a result, rats exhibited REM sleep rebound, replicating previous data from our laboratory in drug-free rats [26], [27]. During the recovery period, all animals exhibited REM sleep rebound, but MET-treated rats had a negative rebound of NREM sleep (e.g., below baseline levels), resulting in less total sleep time than SAL-treated rats. As expected, metyrapone, an inhibitor of 11β-hydroxylase, prevented the increase in CORT plasma levels induced by REM sleep-deprivation, and maintained these levels close to basal. Surprising, however, was the significant difference of plasma CORT levels between control and REM sleep-deprived rats treated with corticosterone. If REM sleep-deprivation increases CORT secretion [12], [36], [45], [46], why additional administration of CORT would lead to lower plasma levels in REM sleep-deprived rats? Induction of feedback inhibition is unlikely to explain this reduction, because both groups received the same dose of corticosterone. The answer to this question may lie on the property of prolonged stress and/or prolonged corticosterone treatment to induce the activity of 11-β-hydroxysteroid dehydrogenase (11β-HSD). This enzyme presents two isoforms: 11-β-HSD types 1 and 2 (11β-HSD1 and 11β-HSD2, respectively) and each one is associated with different corticosterone metabolic fates. 11β-HSD1 catalyzes the inter-conversion of active cortisol to inert cortisone and vice-versa, whereas 11β-HSD2 converts corticosterone to its inactive 11-keto metabolite [47]. Both stress and corticosterone administration induce the activity of 11β-HSD2 [48], [49] and we raised the possibility that combination of both, as occurred in our protocol, could lead to a stronger effect than each manipulation alone. This hypothesis, however, has still to be tested.

The ACTH profile was the opposite to that of CORT, reflecting the effects of the negative feedback mechanism. Thus, metyrapone, which inhibits CORT synthesis, leads to increased CRH and ACTH levels, thus being considered a pharmacological stressor [50]. In addition, metyrapone also increases glucose plasma levels and activates the expression of Fos in several brain structures, including frontal cortex, amygdala and thalamic and hypothalamic nuclei [51]. On the contrary, corticosterone administration increased CORT levels, albeit less so in REM sleep-deprived than in control rats, stimulating the negative feedback, which led to low levels of ACTH.

Repeated metyrapone treatment prolonged waking bouts during sleep deprivation, and yet, there was an impairment of NREM sleep rebound in the first light period of recovery. Single administration of this drug has also been shown to increase waking time, by reducing NREM and REM sleep times, but in this case, the sleep impairment is followed by a homeostatic sleep rebound [19]. The effects hereby reported could be explained by increased CRH activity resulting from sustained blockade of corticosterone synthesis. Numerous evidence supports this idea: CRH i.c.v. administration in rats reduces pentobarbital-induced sleep and increases neuronal excitability; the former being reversed by pre-treatment with anti-CRH antibody [52]. Mice trained to escape a shock exhibit REM sleep rebound, which is blocked by i.c.v. infusion of CRH [6]; CRH receptor-1 (CRH-R1) knock-out mice do not exhibit as intense REM sleep blockade as control mice in response to CRH i.c.v. infusion [53]. Indeed, these receptors appear to be responsible for the sleep impairing effects of CRH, since administration of their antagonist, R121919, improves NREM sleep in an animal model of depression [8] and in depressive patients [54]. CRH receptors are located in thalamic neurons [55], [56] and they inhibit spontaneous activity of reticular neurons [57], which are responsible for synchronization of cortical low frequency EEG seen during NREM sleep [58]. Finally, REM sleep-deprived rats repeatedly treated with CRH also exhibit impairment of sleep homeostasis, with shorter bouts of REM sleep than vehicle-treated rats [35] and reduced power spectrum of low frequency bands (1.0 to 6.0 Hz) [59]. Therefore, increased CRH activity induced by repeated metyrapone treatment could explain the reduction in NREM and REM sleep rebound observed in the present study.

Corticosterone-treated rats also exhibited less NREM sleep during the entire sleep deprivation period. In non-REM sleep-deprived rats, corticosterone doses similar to the one employed in the present study causes a major reduction of NREM sleep for up to 24 h after hormone administration [14]. This effect seems to be dependent on the glucocorticoid concentration acting upon GABA_A_ receptors, since either adrenalectomy or dexamethasone reduces, whereas moderate levels increase the affinity of these receptors [60]. GABA, in turn, inhibits thalamic-cortical connections, resulting in generation of EEG synchronized activity, including spikes and slow waves [58], [61], [62], [63]. Importantly, the thalamus, and its reticular nucleus, is rich in type II glucocorticoid receptors [64], [65], [66], thus, being a natural target for these steroids. Despite NREM sleep inhibition, CORT-treated rats showed a robust increase of SWA and cumulated SWA from the second deprivation day on. SWA during NREM sleep is directly correlated with sleep intensity and is a marker of homeostatic sleep recovery [67]. A recent study, however, claims that sleep deprivation-induced corticosterone secretion is not associated to increased SWA during sleep recovery [68]. Still, corticosterone appears to be, at least in part, involved because adrenalectomy reduces 1.0 to 4.0 Hz power, which is reversed by low levels of corticosterone [15]. Interestingly, adrenalectomy also reduces brain glycogen, which is restored by hormone replacement [69]; brain glycogen is directly related with EEG power [70], [71] and one of the functions attributed to sleep is to restore brain glycogen levels after prolonged periods of waking [72]. Delta activity in NREM sleep accumulates and increases during the course of wakefulness (including forced wakefulness/sleep deprivation), being dissipated within the first hours of the recovery period [24], [73], [74]. In fact, in CORT-treated rats, the observed increase in SWA took place during the sleep deprivation period and in the first hours of recovery (light phase, data not shown). Despite the negative NREM sleep rebound observed during the recovery in MET-treated rats, some studies indicate that total or partial sleep deprivation of various lengths also result in negative NREM sleep rebounds following a small initial positive rebound [28], [75], [76]. We have reason to believe that this effect on NREM sleep is not only due to the homeostatic pressure for REM sleep, but rather the result of the pharmacological manipulation herein employed, since saline-treated sleep-deprived rats did not exhibit the negative NREM rebound.

Corticosterone treatment increased the number and length of REM sleep episodes during sleep recovery. Activation of type II glucocorticoid receptors by this steroid activates type 2 pro-convertase that cleaves pro-opiomelanocortin (POMC) to corticotropin-like intermediate lobe peptide (CLIP) [77], [78], [79], which has a well-established role in prolonging REM sleep episodes [80], [81], [82]. In a previous study we showed that REMSD associated with repeated stress resulted in REM sleep episodes that were two to three times longer than baseline. This procedure also led to corticosterone levels that were intermediate between control animals that were not manipulated (lowest levels) and non-deprived animals submitted to the repeated stress (highest levels) [12]. Glucocorticoid influence on REM sleep follows an inverted U shape curve, with very low or very high levels resulting in impairment, whereas optimal concentrations lead to increase of this sleep phase [83], [84].

In conclusion, the present study showed that either high (corticosterone treatment) or low (metyrapone treatment) circulating levels of corticosterone appear to be detrimental to sleep recovery following 96 h of REM sleep-deprivation. These results reinforce the notion that adequate stress response, and consequently, glucocorticoid levels, is essential for maximal expression of sleep rebound (both at the macro- and micro-structure levels), which, in turn, is thought to be part of the behavioral repertoire necessary for full recovery after stressful situations [85], [86], [87], [88].
